# Aftermath of traumatic tympanic membrane perforation: Our findings at a tertiary care hospital in Pakistan

**DOI:** 10.12669/pjms.37.3.3923

**Published:** 2021

**Authors:** Fazal I Wahid, Muhammad Saleem, Raza Muhammad, Muhammad Riaz Khan

**Affiliations:** 1 Dr. Fazal I Wahid, FCPS. Department of E.N.T, Head & Neck Surgery, Medical Teaching Institute (MTI), Lady Reading Hospital (LRH), Peshawar, Khyber Pakhtunkhwa, Pakistan; 2 Dr. Muhammad Saleem, MCPS, FCPS. Department of E.N.T, K.M.U-I.M.S (KIMS), Kohat, Khyber Pakhtunkhwa, Pakistan; 3 Dr. Raza Muhammad, FCPS. Gajju Khan Medical College Swabi, Khyber Pakhtunkhwa, Pakistan; 4 Muhammad Riaz Khan, FCPS. Naseerullah Babar Memorial Hospital, Kohat Road, Peshawar, Khyber Pakhtunkhwa, Pakistan

**Keywords:** Rupture, Tympanic Membrane, Hearing Loss, Myringoplasty

## Abstract

**Objectives:**

To determine the outcome of traumatic tympanic membrane perforation in a teaching hospital at Peshawar, Pakistan.

**Methods:**

This prospective observational study was performed in the Department of ENT and Head and Neck Surgery, MTI/LRH, Peshawar, Pakistan from January 2016 to December 2019 after approved from Institutional Research and Ethical Board. All consented patients fulfilling inclusion criteria were enrolled. Sample size was calculated 114 using online sample size calculator (OpenEpi). Every patient was evaluated properly, subsequently otoscopy and PTA was performed. Required information was recorded and analyzed using SPSS (version 25).

**Results:**

Out of 114 patients 81 (71.1%) were males and 33(28.9%) were female with male: female ratio of 2.5:1. Patient’s age ranged from 6- 55 years with mean ± SD age of 26.41 ± 9.7 years. Majority of Patients, 46.5%( 53) were in 2^nd^ decade. Amongst the causes slap outnumbered (80, 70.2%), and left ear was involved predominately (67, 58.7%) affecting mainly anterio-inferior quadrant (50, 43.9%). Small size perforation was most common finding (64, 56.1%). Majority of patients (72, 63.2%) presented in a week time and single perforation was the commonest (107, 93.%). Most of traumatic TM perforation got healed spontaneously (97, 85.1%).

**Conclusion:**

It is concluded that spontaneous healing of traumatic membrane perforation occurs in more than 90% cases provided the ear is kept dry and not accompanied by unfavorable conditions.

## INTRODUCTION

Human ear being most exposed part of the body is more prone to any environmental traumatic assault. Trauma to the ear can be isolated or associated with polytrauma. Tympanic Membrane (TM) is a delicate structure made of three layers making lateral boundary of middle ear cleft. Tympanic Membrane separates middle ear from external ear and also contributes in conduction of sound waves from external ear to inner ear.^1^

The different etiological factors for Traumatic Tympanic Membrane Perforation (TTMP) include blast injuries, accidents, firearm injuries, interpersonal violence, iatrogenic and self inflicted injuries. TTMP is a common clinical condition experienced by an otolaryngologist with an estimated incidence of 6.8/ 1000 persons.^2^

The pressure wave hitting the TM results in disruption of its layers. The damage of the TM is directly proportional to the intensity of pressure waves. Pars tensa is the least resistant part of the TM which is commonly involved. In majority of cases there is single perforation in one ear but in case of severe trauma there may be multiple perforations and both ears can be affected.

Symptomotology of TTMP includes hearing loss, pain, bleeding, tinnitus and unsteadiness in case of severe trauma affecting inner ear.^1^ Spontaneous healing of TTMP can occurs in 70 to 90% cases provided the ear is kept sterile and no unfavorable factors are accompanied. As spontaneous healing of TTMP may take place in few months time, thus conservative treatment is treatment of choice. However, if no spontaneous healing occurs then surgical intervention in form of myringoplasty or tympanoplasty are performed. The mechanism of healing of perforation involves proliferation and migration of keratinized squamous epithelium at outer layer of TM. If due to any reason this process does not take place within six months it will result in persistence of perforation demanding surgical intervention.^3^

According to duration TTP perforation can be classified as acute (< 3 months) and chronic (> 3 months). The size of perforation can be calculated as: a. Percentage of perforation = Perforation/ Total area of TM × 100%, so Group I(Small)- area of 0-8 mm^2^, Group II(Medium)-area of 8.1 -30mm^2^, Group III (Large) - area ≥ 3.1mm^2^. b. Percentage of perforation =Perforated Area/ Total Perforated area × 100, thus it is categorized as small (Perforated area < 25% or 1 quadrant), Medium (Perforated area 25% - 50% or two quadrants) and large (Perforated Area 50% -75% or more than two quadrants).^4^

In the past different surgical techniques and various biological materials have been exercised by different people for closure of TTMP. Banzer used pig bladder in 1640, Toynbee applied in 1853 a rubber -like substance, followed by Blake using paper disk in 1887. Camnitz and Bost used paper patch in 1985.^5^

TTMP is quite frequent occurrence in today’s rapidly growing population. As spontaneous healing is a cost effective modality of treatment than surgical intervention with inherited complication. There is paucity of sufficient literature on TTM perforation in developing countries specifically in Pakistan. This study was aimed to look into outcome of traumatic TM perforation in our setting.

## METHODS

This prospective observational hospital based study was performed in the department of Ear, Nose, throat and Head and Neck Surgery, Medical Teaching Institute (MTI)/Lady Reading Hospital (LRH), Peshawar, Khyberpakhtunkhwa (KPK), Pakistan from January 2016 to December 2019 after getting ethical approval from Institutional Research and Ethical Board. (Ref: 1106/MA, Dated: 01-01-2016). All consented patients fulfilling inclusion criteria were enrolled. All those patients sustaining traumatic tympanic membrane perforation irrespective of etiology, duration and gender were included in the study, while patients having non-traumatic TM perforation, not willing for study or those lost from follow up were excluded. Sample size was calculated 114 using online sample size calculator (OpenEpi: Sample Size for X-Sectional, Cohort, and Clinical Trials, taking confidence interval of 95% and the margin of error of 5% https://www.openepi.com/Menu/OE_Menu.htm.)^6^ Convenient (Non-probability) sampling technique was exercised.

Every patient was thoroughly evaluated in terms of detail history, meticulous examination and relevant investigations. Patient was inquired about occurrence, duration, event site, nature and associated other organ injury. Otoscopic examination was performed focusing on side, location, size, number, margins of perforation and associated injuries. Tuning fork tests were carried out. Pure Tone Audiometry (PTA) was accomplished by same senior audiometrician using standard two channel clinical audiometer (Amplaid 455Itly). PTA was recorded for the frequencies of 0.5,1,2,4 kHz. These patients were scheduled for monthly follow up till six months minimum to look for spontaneous healing of TM perforation or otherwise.

All required information was recorded on a predesigned proforma and was analyzed through SPSS (version 25). Mean ± Standard deviation (SD) and frequency and percentage were computed for Quantitative and Qualitative variables respectively. Confidence interval (CI) was taken as 95% and the margin of error was accepted as 5%, while P-value <0.05 was taken significant.

## RESULTS

**Fig.1:**
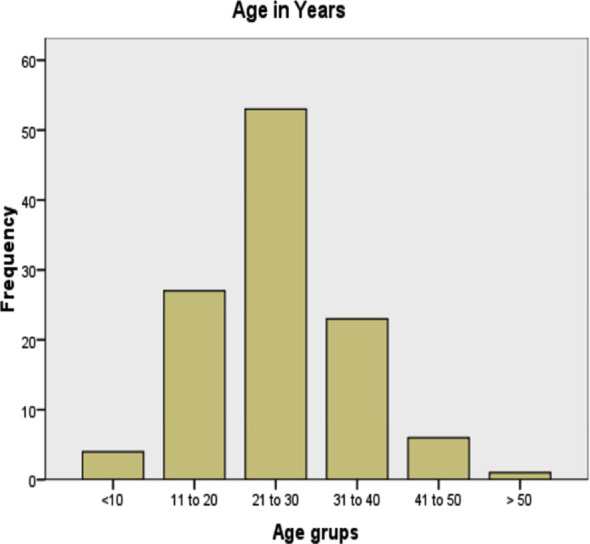
Bar chart showing age of the patients in years.

In this study out of 114 patients 81 (71.1%) were males and 33(28.9%) were female with male: female ratio of 2.5:1. Patient’s age ranged from 6- 55years with mean ± SD age of 26.41 ± 9.7 years. Majority of Patients, 46.5% (53) were in 2^nd^ decade followed by 1^st^ and 3^rd^ decade of life 23.7% (27) and 20.2% (23) respectively (Fig.1). Area wise distribution of the patients was such that 45.6% (52) belonged to Peshawar district, 23.7% (27) to northern districts of KPK, 21.1% (24) to southern districts of KPK and 6.1% (7) to other provinces and only 3.5% (4) were from Afghanistan.

We observed that slap outnumbered (80, 70.2%), amongst the causes of TTMP followed by blast (17, 14-9%). Left ear was involved predominately (67, 58.7%) while in majority of cases (50, 43.9%) anterio-inferior compartment of the TM was affected. Small size perforation was most common finding (64, 56.1%). Majority of patients (72, 63.2%) presented to ENT clinic within a week time and single perforation was the commonest observation (107, 93%) (Table-I). Conductive type hearing loss was common (94, 82.5%), however mild degree of hearing loss was recorded in majority of patients (59, 51.8%). Most of traumatic TM perforation got healed spontaneously (97, 85.1%), while overlay myringoplasty was commonest (7, 6.1%) procedure performed in those where perforation persisted beyond six months (Table-II).

**Table-I:** Demographic data of Patients with Traumatic Tympanic Membrane Perforation (n=114).

Cause of Traumatic Tympanic Membrane Perforation		

Item	Frequency	Percent (%)
Slap	80	70.2
Blast	17	14.9
Fire Arm Injuries	4	3.5
Self inflicted	6	5.3
Suction/ Probing/syringing	7	6.1
Total	114	100.0
***Side of Traumatic Tympanic Membrane Perforation***		
Right ear	46	40.3
Left ear	68	59.6
Total	114	100.0
***Site of Traumatic Tympanic Membrane Perforation***		
Anterio-superior	21	18.4
Anterio-inferior	50	43.9
Posterio-superior	11	9.6
Posterio-inferior	32	28.1
Total	114	100.0
***Size of Traumatic Tympanic Membrane Perforation***		
Small	64	56.1
Medium	45	39.5
Large	5	4.4
Total	114	100.0
***Duration of Traumatic Tympanic Membrane Perforation***		
< a week	72	63.2
< a month	35	30.7
> a month	7	6.1
Total	114	100.0
***Number of Traumatic Tympanic Membrane Perforation***		
Single	107	93.9
Multiple	7	6.1
Total	114	100.0

**Table-II:** PTA Findings, Degree of hearing loss and outcome of Traumatic Tympanic Membrane Perforation (n=114).

Pure Tone Audiometry(PTA) findings		

Item	Frequency	Percent (%)
Conductive Hearing Loss	94	82.5
Sensorineural Hearing Loss	17	14.9
Mixed Hearing Loss	3	2.6
Total	114	100.0
***Degree of hearing Impairment***		
Mild Hearing Loss	59	51.8
Moderate Hearing Loss	40	35.1
Severe Hearing Loss	15	13.2
Total	114	100.0
***Outcome Traumatic Tympanic Membrane Perforation***		
Spontaneous Healing	97	85.1
Overlay Myringoplasty	7	6.1
Underlay Myringoplasty	3	2.6
Tympanoplasty Type I	4	3.5
Tympanoplasty Type II	2	1.8
Tympanoplasty Type III	1	.9
Total	114	100.0

Traumatic tympanic membrane was significantly common on left side affecting pars tensa of the ear drum (p<0.05), however there was no effect of size, duration and severity of hearing loss on spontaneous healing of traumatic membrane perforation (p>.05).

## DISCUSSION

Trauma to human body is on rise due to rapid growth in technology. Traumatic tympanic membrane perforation may cause difficulty in hearing that needs to be treated on priority before any complication. In this study males predominates with male: female ratio of 2.5:1, that simulates to most of the studies but contradicts to some studies.^1^,^2^ In our study majority of patients were in 2^nd^ decade of life (46.5%, 53) with mean ± SD age of 26.41 ± 9.7 years (Age ranged 6-55 Years), which coincides that of Ahluwalia from India with mean age of 27.9 years and maximum patients (45.0%) in second decade of life.^7^ The mean age and commonest age of presentation of this study is also comparable with results of Al-Juboori from Iraq and Gao and colleagues from china and some other studies.^8^,^9^

In current study amongst the cause of TTMP, slap trauma was most common (70.2%, n-80), subsequently trauma due to blast was found in 17 patients (14.9%), which is keeping with results of Ahluwalia, Al-Juboori, Singh, Irfan, Rai, who also reported that TTMP was mainly due to slap trauma to the ears.^7^,^8^,^10^-^12^ However etiologic factors of this study differs from that of, Ravi, Dawood, Sannigrahi, and Al-Obiedi where traumatic tympanic perforation was predominantly caused by blast injuries while in one study road traffic accidents was main cause of tympanic membrane perforation.^3^,^5^,^13^,^14^

We found that in majority of patients left ear was affected more (58.7%, n-67), and commonest site of perforation of membrane was anterio-inferior (43.9% n-50) quadrant. Similarly, Adegbiji and colleagues from Nigeria conducted a study in 2018 and their observations were that left ear was involved more than right (43.9%, 232), grade 1(small size) perforation was most common (39.3%, n-208) and central part of membrane was predominantly affected (38.2%, n-202).^15^ These results are also supported by studies of Sogebi and Wani.^16^,^17^ The left ear predominance can be explained as during interpersonal assault left is more close to assaulter.

Majority of patients (72, 63.2%) presented to ENT clinic within a week time and single perforation was the commonest observation (107, 93%), which simulates to study of Sogebi, where patients presented to clinic between few hours to 23 days with mean duration after injury was 3 days.^16^ Similarly, Wani from India carried out study in 2016 on traumatic TM perforation and he also observed that 323 (92.3%) patients had single perforation while 27 (7.7%) had multiple perforations.^17^

In current study conductive hearing loss was more common (94, 82.5%), however mild degree of hearing impairment was recorded in 59 patients (51.8%). Likewise in Singh’s study conductive hearing loss was found in 50 patients (83.33%) while mild degree of hearing loss was noted in 40 patients (66.66%).^10^ Our observation is also in accordance with study of Adegbiji from Nigeria, revealed that conductive hearing loss was the most common type of hearing impairment accounted for 326(61.6%), followed by sensorineural hearing loss 134(25.3%), while the common degree of hearing impairment was mild to moderate degree of hearing loss that accounted for 249(47.1%).^15^

In present study spontaneous healing took place in 97 patients (85.1%), however amongst surgical procedures overlay myringoplasty was commonest (7, 6.1%) in those where perforation persisted beyond six months, which is keeping with Wani’s work where overall spontaneous healing was achieved in 91.1% patients, and tympanoplasty was performed in 8(2.3%) patients.^17^ According to available literature spontaneous healing of TTMP occurs in 80 – 100% cases.^5^,^10^-^16^,^18^,^19^

As slap on the ear was more common resulting perforation at anterio-inferior quadrant of TM on left side hence there was statistically significant association between cause of trauma and site and side of perforation (x=24.13, p=0.020), (x=57.10.p=0.000), that is supported by study of Sannigrahi who found significant association *between mode of injury and tympanic membrane perforation after three months (χ2 = 23.30; p = .00001)*.^13^ Another study results also showed that there was statistically significant difference between TM perforation and age of the patient, severity of deafness, size of perforation (p <0.05) as shown.^8^

Traumatic tympanic membrane was significantly common on left side affecting pars tensa of the ear drum (p<0.05), however there was no effect of size, duration and severity of hearing loss on spontaneous healing of traumatic membrane perforation (p>.05).

Similarly, Al-Juboori confirmed that there was no statistically significance difference between healing of TM perforation and gender of the patient, laterality and causes of the Injury (p>0.05).^8^

### Limitation:

Limitation of the study was that sample size was small and further study on large sample size with long follow up is required to know the long term outcome of traumatic tympanic membrane perforation.

## CONCLUSION

It is concluded that spontaneous healing of traumatic membrane perforation occurs in more than 90% cases provided the ear is kept dry and is not accompanied by unfavorable conditions. Those cases which do not heal spontaneously needs myringoplasty for better outcome.

### Authors’ Contribution:

**FIW:** conceived, designed and did statistical analysis & editing of manuscript and is responsible for accuracy and integrity of the research work.

**MS, MR:** did data collection and manuscript writing.

**MRK:** did review and final approval of manuscript.
